# Cellular response to alkylating agent MNNG is impaired in STAT1‐deficients cells

**DOI:** 10.1111/jcmm.12887

**Published:** 2016-07-27

**Authors:** Laurent Ah‐Koon, Denis Lesage, Elodie Lemadre, Inès Souissi, Remi Fagard, Nadine Varin‐Blank, Emmanuelle E. Fabre, Olivier Schischmanoff

**Affiliations:** ^1^ INSERM, U978BobignyFrance; ^2^ Université Paris 13, UFR SMBH, Sorbonne Paris CitéLaboratoire d'excellence INFLAMEXBobignyFrance; ^3^ AP‐HP, GHU‐PSSDHôpital Avicenne, Service de BiochimieBobignyFrance

**Keywords:** MMR, c‐Abl, STAT1, p53, MNNG

## Abstract

The S_N_1 alkylating agents activate the mismatch repair system leading to delayed G_2_/M cell cycle arrest and DNA repair with subsequent survival or cell death. STAT1, an anti‐proliferative and pro‐apoptotic transcription factor is known to potentiate p53 and to affect DNA‐damage cellular response. We studied whether STAT1 may modulate cell fate following activation of the mismatch repair system upon exposure to the alkylating agent *N*‐methyl‐*N*′‐nitro‐*N*‐nitrosoguanidine (MNNG). Using STAT1‐proficient or ‐deficient cell lines, we found that STAT1 is required for: (*i*) reduction in the extent of DNA lesions, (*ii*) rapid phosphorylation of T68‐CHK2 and of S15‐p53, (*iii*) progression through the G_2_/M checkpoint and (*iv*) long‐term survival following treatment with MNNG. Presence of STAT1 is critical for the formation of a p53‐DNA complex comprising: STAT1, c‐Abl and MLH1 following exposure to MNNG. Importantly, presence of STAT1 allows recruitment of c‐Abl to p53‐DNA complex and links c‐Abl tyrosine kinase activity to MNNG‐toxicity. Thus, our data highlight the important modulatory role of STAT1 in the signalling pathway activated by the mismatch repair system. This ability of STAT1 to favour resistance to MNNG indicates the targeting of STAT1 pathway as a therapeutic option for enhancing the efficacy of SN1 alkylating agent‐based chemotherapy.

## Introduction

The monofunctional S_N_1 alkylating agent *N*‐methyl‐*N*′‐nitro‐*N*‐nitrosoguanidine (MNNG), has strong mutagenic and carcinogenic properties that induce cell cycle arrest, DNA repair and/or apoptosis. The main mutagenic and cytotoxic alteration created by MNNG is formation of O^6^‐methyl guanine (O^6^MeG) inducing thymine mispair during DNA replication. Two DNA repair systems might then intercede. First, O^6^‐methylguanine‐DNA methyl transferase (MGMT) catalyses demethylation counteracting the lethal effects of O^6^MG 1. Second, the mismatch repair (MMR) system, an evolutionary conserved multiproteic complex, participates in the recognition and the repair of mispaired nucleotides 2.

Mismatch repair proteins form major heterodimers MSH2/MSH6 (hMutSα) and MLH1/PMS2 (hMutLα). hMutSα preferentially recognizes base‐pair mismatches and short insertion/deletion mispairs while hMutLα activates downstream signalling events [reviewed in 3]. hMutSα also detects O^6^MeG DNA adducts produced by S_N_1 DNA methylators 4. Mismatch repair system activation by extensive DNA alkylation drives processes leading to DNA double strand breaks 5 due to simultaneous processing of clustered apurinic/apyrimidinic sites on opposite DNA strands 6. Mismatch repair complexes respond to exposure to S_N_1 DNA methylators leading to a delayed accumulation of cells in G_2_/M phase occurring after the second S‐phase 7. The next step consists of a cellular balance between DNA repair and survival, or conversely, cell death. Indeed, cells with defects in hMutSα or hMutLα heterodimers fail to elicit a G_2_/M checkpoint response and are defective in cell cycle arrest and apoptosis upon treatment with S_N_1 methylators causing alkylation tolerance 8.

Upon O^6^MeG adducts detection, hMutSα and hMutLα complexes constitute a scaffold for the recruitment of several partners including Ataxia Telangiectasia Mutated (ATM), Ataxia Telangiectasia and Rad3‐Related Protein (ATR), Checkpoint Kinase (CHK)1, Checkpoint Kinase (CHK)2, p53 and Abelson Murine Leukaemia Viral Oncogene Homolog 1 (c‐Abl) at the sites of DNA alkylation 9, 10, 11, 12. Cellular responses to DNA double strand breaks involve ATM/CHK2 signalling [reviewed in 13]. Although activation of ATM/CHK2 pathway leads to p53 stabilization 12, 14, MNNG‐induced G_2_/M arrest and apoptosis are triggered by both p53‐dependent and ‐independent pathways 13, 15. Interestingly, important roles for the tyrosine kinase c‐Abl have been recently established in MMR‐induced signalling. Indeed, MNNG‐induced MMR‐dependent G_2_/M arrest and apoptosis are also controlled through hMLH1/c‐Abl/p73α/Growth Arrest and DNA‐Damage‐Inducible Protein (GADD45α) signalling. Interestingly, the tyrosine kinase activity of c‐Abl is essential to these responses to DNA damage, which are mediated by the p53‐homologue, p73 16, 17.

How MMR‐dependent G_2_/M cell cycle arrest may lead either to cell survival or to cell death remains elusive. Modulators involved in these processes – such as p53 or c‐Abl – may be critical to determine cell fate following DNA damage. A potential regulatory candidate is signal transduction and activator of transcription (STAT)1, the main effector of IFNγ in innate immunity 18, 19. At the molecular level, phosphorylation of STAT1 and subsequent nuclear translocation induces differential transcriptional regulation of anti‐proliferative and pro‐apoptotic genes 20. STAT1 up‐regulates expression of cyclin‐dependent kinase inhibitors p21^waf1^ and p27^kip1^ and inhibits expression of various cyclins and of c‐myc 21, 22.

STAT1 is also involved in the modulation of DNA damage‐induced cell death. In murine embryonic fibroblasts, apoptosis induced by DNA damaging agents such as doxorubicin or cisplatin requires a functional STAT1. In these cells, STAT1 potentiates p53‐dependent transcription of target genes Noxa, Bax and Fas 23. In B cells, STAT1 physically interacts with p53, modulates p53 activity and sensitizes the cells to fludarabine‐induced apoptosis 24. Reciprocally, recent data indicate that a treatment of EBV‐transformed B cells with doxorubicin and fludarabine, activates STAT1 in a p53‐dependent manner and that STAT1 interacts both with p53 and c‐Abl 25. The significant role of STAT1‐p53 interaction in regulating p53 death responses is supported by the fact that STAT1 deletion enhances tumour development in p53‐null mice 26.

Since p53 and c‐Abl are important mediators of MMR‐induced signalling, we sought to determine whether STAT1 modulates the signalling pathways induced by the MMR system in MNNG‐treated cell lines. Using cells proficient or deficient for STAT1, but expressing p53 and c‐Abl, we found that STAT1 is required for efficient DNA repair, G_2_/M checkpoint overpass and long‐term survival following treatment with MNNG. This process implies a DNA binding complex comprising p53, MLH1, and more importantly, depending on the presence of STAT1, the recruitment and the kinase activity of c‐Abl. Thus, our results place STAT1 as a major contributor in cell survival following DNA alkylation.

## Materials and methods

### Reagents

*N*‐methyl‐*N*′‐nitro‐*N*‐nitrosoguanidine; TCI, Zwijndrecht, Belgium) was prepared in absolute ethanol. Cisplatin was obtained from Merck Chemical (Fontenay‐sous‐Bois, France). Human recombinant γ‐IFN (#300‐02) was obtained from Peprotech (Neuilly‐sur‐Seine, France).

### Antibodies

Anti‐tyrosine‐701‐phosphorylated‐STAT1 (#9167), anti‐STAT3 (#4904), anti‐MLH1 (#4256), anti‐ATM (#2873), anti‐serine‐1981‐phosphorylated‐ATM (#5883), anti‐CHK2 (#6334), anti‐threonine‐68‐phosphorylated‐CHK2 (#2197), anti‐ATR (#13934), anti‐serine‐428‐phosphorylated‐ATR (#2853), anti‐CHK1 (#2360), anti‐serine‐345‐phosphorylated‐CHK1, anti‐HSP90 (#4874), anti‐serine‐139‐phosphorylated‐H2AX (#9718), anti‐c‐ABL(#2862), anti‐p53 (#9282), anti‐serine‐15‐phosphorylated‐p53 (#9284) and anti‐MSH2 (#2017) were purchased from Cell Signaling Technology (Ozyme, Montigny‐le‐Bretonneux, France). Anti‐STAT1α (#SC‐345), anti‐STAT1α and STAT1β isoforms (#SC‐346) and anti‐α‐actin (#SC‐1616) were from Santa Cruz/Tebu‐Bio (Le‐Perray‐en‐Yvelynes, France). Anti‐α‐tubulin (#T8203) was from Sigma‐Aldrich (Saint‐Quentin‐Fallavier, France).

### Cell culture

The STAT1‐proficient (STAT1^+/+^, 2C4) and STAT1‐deficient (STAT1^−/−^, U3A) human fibrosarcoma cell lines were kindly provided in 2010 by Dr S. Boisson‐Dupuis (INSERM U980). They both originate from HT1080 human sarcoma cell line and have been described in (Mc Kendry *et al*. 1991 27). Cells lines were cultured in DMEM supplemented with 10% foetal calf serum, 100 U/ml penicillin, 10 μg/ml streptomycin (PAA Laboratories, Les‐Mureaux, France) at 37°C in a humidified 5% CO_2_ atmosphere. Cells were repeatedly tested for STAT1 expression during the course of the study by Western blotting or flow cytometry.

### Proliferation curves of STAT1^−/−^ and STAT1^+/+^ cells

Cells were plated in 12‐well plates at day 0 at a concentration of 10^4^/ml. The number of viable cells was counted at 24, 48 and 72 hrs. Doubling time were calculated using the formula: [Time * Ln 2/[Ln (C2/C1)], where C1 is the initial cell concentration and C2 the cell concentration after 48 hrs.

### Generation of STAT1^−/−Tα^ cell line, stably expressing STAT1‐alpha

STAT1^−/−Tα^ cell line was obtained from STAT1^−/−^ cells transfected with pRc/CMV STAT1‐alpha expression plasmid (generous gift of Dr J. Darnell, Rockefeller University, NY, USA) using standard calcium/phosphate methodology. Stably transfected cells were selected using 1 mg/ml G418 resistance (PAA Laboratories). Selected clones were isolated, maintained in G418‐containing medium and controlled for STAT1 expression using western blotting.

### Cell lysis

Cells were washed in PBS, lysed in NRSB minus buffer (62.5 mM Tris pH 6.8, SDS 2%, glycerol 10%). For oligonucleotide pulldown experiments, cytoplasmic and nuclear extracts were collected using Panomics Nuclear Extraction kit (Ozyme, Saint‐Quentin‐en‐Yvelines, France). Protein quantification was measured using Pierce BCA protein assay kit (Thermo Fisher, Illkirch, France).

### Mitochondrial dehydrogenase activity and cell viability

The CellTiter 96 AQ_ueous_ Non‐Radioactive Cell Proliferation Assay – MTS (Promega, Charbonnières‐les‐Bains, France) was performed following the manufacturer instructions. For MNNG treatment, cells were exposed to 10 μM MNNG for 1 hr, washed twice with PBS and further incubated in MNNG‐free medium at 37°C. For cisplatin treatment, cells were incubated continuously with 40 μM of the genotoxic agent. Supernatants were collected at 24, 48 or 72 hrs, and absorbances were measured at 492 nm on a Thermo Scientific Multiskan FC luminometer (Thermo Fisher). Cell viability was also assessed using the Trypan blue exclusion method. Dehydrogenase activity and cell viability were measured at 24, 48 or 72 hrs time‐points and calculated as a percentage relative to the activity or number of untreated cells at each time‐point respectively. Three independent experiments were performed.

### Clonogenic assay

Clonogenicity was assessed as described with slight modifications 28. Briefly, cells were seeded (1000 cells per well) in a six‐well plate 8 hrs prior to treatment. *N*‐methyl‐*N*′‐nitro‐*N*‐nitrosoguanidine (0.2–10 μM) was added for 1 hr incubation. After treatment, the medium was removed, cells were carefully washed twice with PBS and fresh medium was added. Ten days after treatment, cells were washed three times in PBS, fixed and stained in v/v ethanol‐methylene blue. Each plate was scanned at high resolution (2400 dpi) with a Perfection V700 photo scanner (Epson, Levallois‐Perret, France). Colonies containing at least 50 cells were counted. Clonogenic survival was assessed as a percentage to control cells that have not been exposed to MNNG. Three independent experiments conducted in triplicate were performed.

### Western blotting

All primary antibodies were used at 1/1000. Western blotting was revealed by chemiluminescence (Amersham, Orsay, France) using a Chemidoc apparatus and the Image Lab 4.2 software (Bio‐Rad, Marnes‐la‐Coquette, France). Signal intensities were measured using ImageJ software (NIH, Bethesda, MD, USA). Ratios were determined relative to the loading control.

### Immunocytochemistry

Cells were seeded (2 × 10^4^ cells per well) in eight‐well Permanox Lab‐Tek plates (Dutscher, Issy‐les‐Moulineaux, France) and incubated with 10 μM MNNG for 1 hr. Cells were further incubated in MNNG‐free medium at 37°C during 24, 48 or 72 hrs. After fixation, cells were permeabilized in PBS buffer containing 0.1% Triton X‐100 for 15 min. Cells were further probed with rabbit anti‐serine‐139‐phospho‐H2AX (#2577; Cell Signaling). Nuclei were counterstained with 4,6‐diamidino‐2‐phenylindole dihydrochloride (DAPI; Sigma‐Aldrich). After three washes, coverslips were mounted with DAKO fluorescent mounting medium (Dako France, Trappes, France). Fluorescent images were acquired using a Leica DM 2000 and analysed with the Leica Application Suite 2.5 software (Leica Microsystems, Nanterre, France).

### Flow cytometry

For STAT1 expression analysis: 2.0 × 10^6^ cells were fixed in BD Phosflow^™^ Lyse/Fix Buffer 1× (BD Biosciences, Le‐Pont‐de‐Claix, France) for 10 min. at 37°C and further permeabilized using BD Phosflow^™^ PERM buffer III (BD Biosciences) following the instructions of the manufacturer. Cells were labelled using the following antibodies: Alexa Fluor^®^ 647 Mouse Anti‐STAT1 (N‐terminus) (#558560) or Alexa Fluor^®^ 647 Mouse IgG1 κ control isotype (#557783; BD Biosciences). Data were collected with a Becton Dickinson FACS Canto II (BD Biosciences) and analysed with BD FACS DIVA Software V6.1.3 (BD Biosciences). For cell cycle analysis, 1.0 × 10^6^ cells were exposed to 10 μM MNNG for 1 hr. Cells were collected at the indicated times after treatment. After fixation in ice‐cold 70% ethanol, cells were recovered by centrifugation and further incubated with RNAse A (1 μg/ml; Thermo Fisher) for 30 min. at room temperature. After three washes, 20 μg/ml propidium iodide (Sigma‐Aldrich) was added and incubated at room temperature in the dark for 45 min. prior to analysis. Data were analysed with FlowJo software (Tree Star Inc., Ashland, OR, USA).

### Oligodeoxynucleotide pull down

Biotinylated oligonucleotide containing the canonical p53 consensus binding sequence (Biotin‐5′‐GACATGCCCGGGCATGTCC‐3′) was obtained from Sigma‐Aldrich. 2 × 10^7^ cells were treated with 10 μM MNNG for 1 hr. 72 hrs after treatment, nuclear protein extracts (300 μg) were incubated with 1 μg of double stranded oligodeoxynucleotide (ODN) for 1 hr at 4°C in a final volume of 50 μl binding buffer [10 mM 4‐(2‐hydroxyethyl)‐1‐piperazineethanesulfonic acid (HEPES) pH 8.0, ethylenediaminetetraacetic acid (EDTA) 100 μM, NaCl 50 mM, KCl 50 mM, MgCl_2_ 5 mM, spermidin 4 mM, 1,4‐dithiothreitol (DTT) 2 mM, bovine serum albumin 0.1 mg/ml, glycerol 2.5%, Ficoll 4%]. The complexes were captured by incubation with Avidin UltraLink Resin (Pierce, Thermo Fisher) for 2 hrs at 4°C. After three washes in a buffer containing: 100 mM NaCl, 10 mM HEPES pH 7.4, the complexes were resuspended in SDS sample buffer (50 mM Tris HCl pH 6.8, 2% SDS, 10% glycerol, 1% β‐mercaptoethanol, 12.5 mM EDTA, 0.02% bromophenol blue), separated on 8% SDS‐PAGE, transferred on a nitrocellulose membrane and processed for western blotting.

### Statistical analysis

Calculations of the mean and standard deviation were performed with Microsoft Excel. Statistical analysis and comparison of each set of experimental means was performed with Graphpad Instat 3.0 (Graphpad Software Inc., La Jolla, CA, USA).

## Results

### Long‐term survival following MNNG exposure requires STAT1

**Figure 1:**
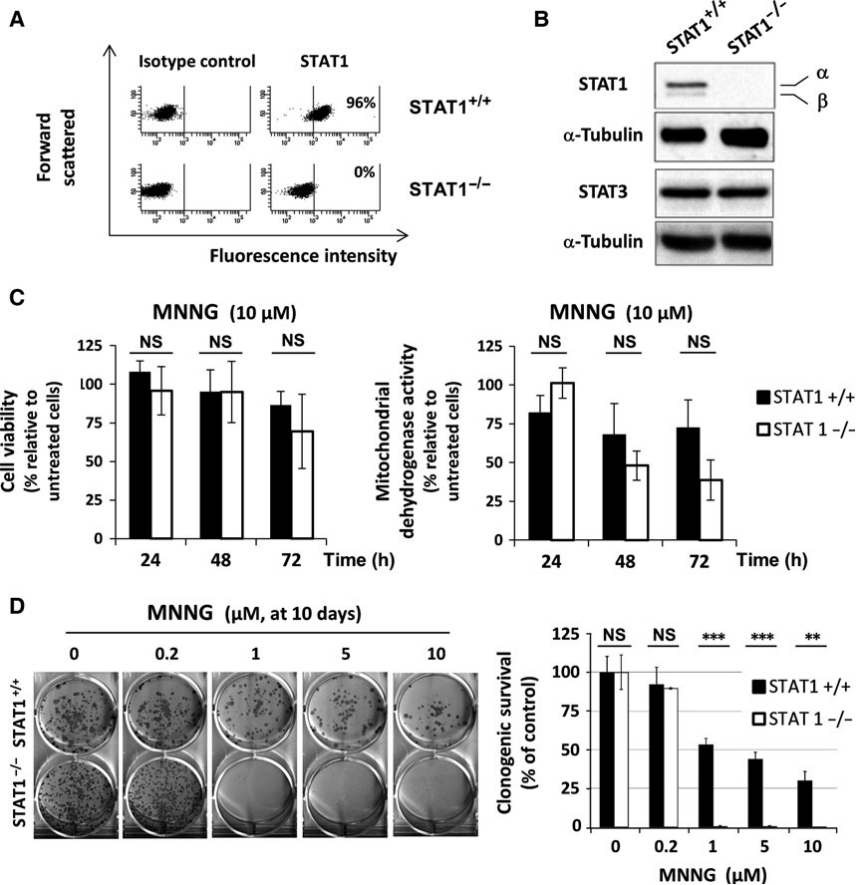
Cellular response to MNNG according to STAT1 expression. STAT1 expression was analysed: (**A**) by flow cytometry (the proportion of cells expressing STAT1 is indicated for each cell line), (**B**) by western blotting (α‐Tubulin and STAT3 were also assayed as controls), (**C**) Cells were exposed to 10 μM MNNG for 1 hr. At the indicated times, cell viability was assessed by Trypan blue vital staining (left panel) and cell dehydogenase activity was analysed by MTS assay (right panel). Results are plotted as percentage relative to untreated cells at the same time‐points. Data are from three independent experiments. Error bars represent the standard deviation (NS, non‐significant). (**D**) Long‐term clonogenic survival was analysed 10 days following a 1 hr exposure to the indicated doses of MNNG. A typical clonogenic assay representative of three independent experiments is shown. Percentage of colonies containing 50 cells or more was evaluated relative to untreated control. Statistical significance was determined by paired *t*‐test (***P < 0.001; **P < 0.01).

We evaluated the participation of STAT1 to the cellular response following DNA damage induced by MNNG in human STAT1‐proficient (STAT1^+/+^) or ‐deficient (STAT1^−/−^) fibrosarcoma cell lines (Fig. 1A and B). Both STAT1^+/+^ and STAT1^−/−^ cell lines exhibited similar expression levels of STAT3 and a functional MMR system assessed by the absence of microsatellite instability (Fig. 1B, Table S1). First, we checked whether a 10 μM MNNG treatment would neither alter STAT1 expression nor its Y701‐phosphorylation and this was substantiated in STAT1 expressing cells (Fig. S1). We then studied cell viability and mitochondrial dehydrogenase metabolic activity in STAT1^+/+^ and STAT1^−/−^ cell lines (Fig. 1C). We first verified in our model the previously demonstrated dependency of Cisplatin cytotoxicity to STAT1 29. Therefore, we controlled that the DNA damaging agent Cisplatin required STAT1 expression to affect metabolic activity as early as 24 hrs (Fig. S2). In marked contrast, the short‐term cytotoxic effect of MNNG was STAT1‐independent. High‐dose exposure to MNNG did not lead to significant differences between STAT1^+/+^ and STAT1^−/−^ cells neither in viability (Fig. 1C, left) nor in metabolic activity (Fig. 1C, right). However, we observed an overall reduction in viability and metabolism starting at 72 hrs and 48 hrs, respectively. Therefore, considering the weak short‐term effect of MNNG, long‐term survival was evaluated using a clonogenic assay. Clonogenic survival following MNNG treatment showed STAT1 dependency in addition to MNNG dose dependency. Ten days following 1 μM MNNG treatment, STAT1^−/−^ cells lost their ability to form colonies while, even at 10 μM MNNG, STAT1^+/+^ cells were still able to form colonies (Fig. 1D). These results suggest that, in our model, while STAT1 did not exhibit a protective effect at short term, it protected cells from long‐term cytotoxicity upon MNNG treatment.

### Persistence of MNNG‐induced DNA double strand breaks is STAT1‐dependent

**Figure 2:**
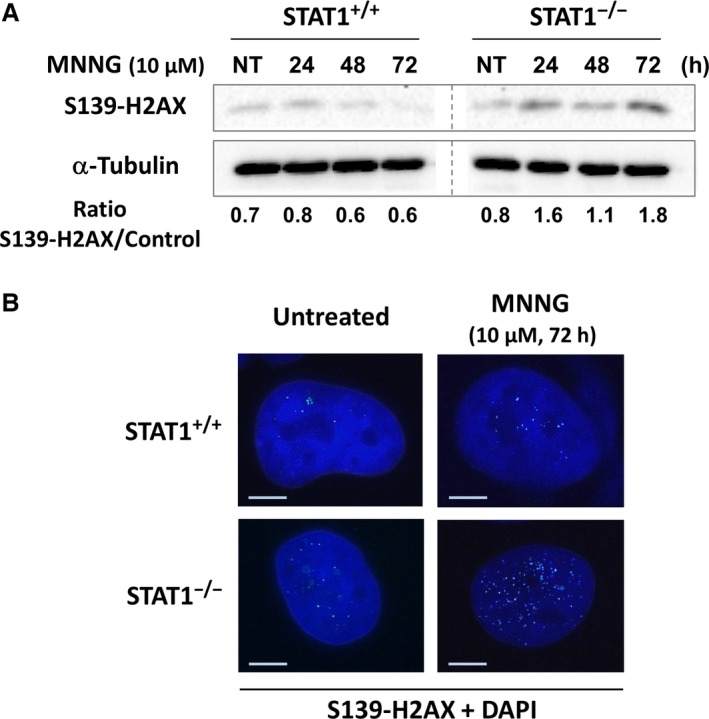
Higher activation of histone H2AX following MNNG exposure in the absence of STAT1. STAT1^+/+^ and STAT1^−/−^ cells were treated for 1 h or not (NT) with 10 μM MNNG. (**A**) Western blot analysis of S139‐H2AX at the indicated time‐points. A typical experiment out of three is shown. α‐Tubulin was used as loading control. Both panels are cut from the same western blot. (**B**) 72 hrs following treatment, cells were also fixed and immunostained for S139‐H2AX. Nuclei were counterstained with DAPI. A typical immunostaining out of two is shown. Scale bars, 5 μm.

To delineate the involvement of STAT1 on long‐term survival in our model, we studied whether STAT1 deficiency might interfere with DNA repair following MNNG during the 72 hrs period while metabolic activity dropped consistently. We thus studied the phosphorylation of histone H2AX on Serine‐139, as a marker of DNA double strand breaks 30. Phosphorylation pattern analysed by western blot indicated a higher activation of H2AX in STAT1^−/−^ cells most significantly at 72 hrs following 10 μM MNNG treatment (Fig. 2A). We further investigated these differences using immunohistochemistry at 72 hrs. Both the proportion of cells with S139‐H2AX labelling and the number of foci per positive cell were strikingly different between STAT1^+/+^ and STAT1^−/−^ cells (49% *versus* 96%, respectively; Fig. 2B and Table 1). Thus, an accumulation of double strand breaks occurred in the absence of STAT1. These data suggest that STAT1 is required for an efficient repair of MNNG‐induced DNA lesions.

**Table 1:** Proportion of cells with activated histone H2AX in STAT1^+/+^ and STAT^−/−^ cells following MNNG exposure

	Percentage of S139‐H2AX‐positive cells/total cells		Number of foci per cell	
	STAT1^+/+^	STAT1^−/−^	STAT1^+/+^	STAT1^−/−^
Untreated	30	38	9	10
MNNG (10 μM, 72 hrs)	49	96^a^	25	55

a Among which 50% exhibiting a very strong staining. S139‐H2AX‐positive cells were numbered relative to a minimum of 80 cells. Numbers of foci per cell were evaluated under similar conditions.

### Cell cycle progression following MNNG exposure depends on STAT1 expression

**Figure 3:**
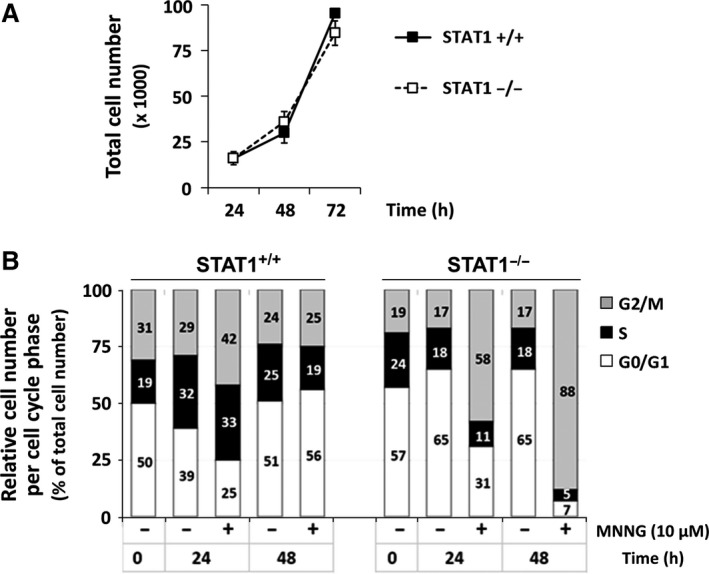
G_2_/M accumulation following MNNG exposure in the absence of STAT1. (**A**) Proliferation curves of STAT1^+/+^ and STAT1^−/−^ cells. Error bars represent the standard deviation. Experiment conducted in triplicate and repeated twice. (**B**) STAT1^+/+^ and STAT1^−/−^ cells were treated or not for 1 hr with 10 μM MNNG. At the indicated times, cells were fixed, stained with propidium iodide and analysed by flow cytometry. Cumulative plots and numbers indicate the relative percentages of cells present in each of the three phases of the cell cycle. A typical experiment out of two is shown.

We then studied whether persistence of MNNG‐induced DNA lesions observed in STAT1‐deficient cells would alter cell cycle progression. Similar doubling times of STAT1^+/+^ (18.6 hrs) and STAT1^−/−^ (19.0 hrs) cell lines indicated that STAT1 deficiency did not alter cell basal growth rate in our model (Fig. 3A). Therefore, cell cycle analysis was performed in STAT1‐proficient and ‐deficient cells at 24 and 48 hrs following a 1 hr exposure to 10 μM MNNG (Fig. 3B). Without any treatment, in STAT1^+/+^ cells the proportion of G_2_/M cells ranged from 31% to 24% throughout a 48 hrs period, whereas it remained below 20% in STAT1^−/−^ cells. Upon MNNG treatment, we observed a striking differential cell cycle progression between STAT1^+/+^ and STAT1^−/−^ cells. The proportion of STAT1^+/+^ cells in G_2_/M phase increased transiently reaching 42% at 24 hrs. In contrast, in STAT1^−/−^ cells, a strong accumulation of the cells in G_2_/M phase was already observed at 24 hrs (58%), reaching levels as high as 88% at 48 hrs.

### Increased CHK2 activation following MNNG exposure in STAT1^−/−^ cells

**Figure 4:**
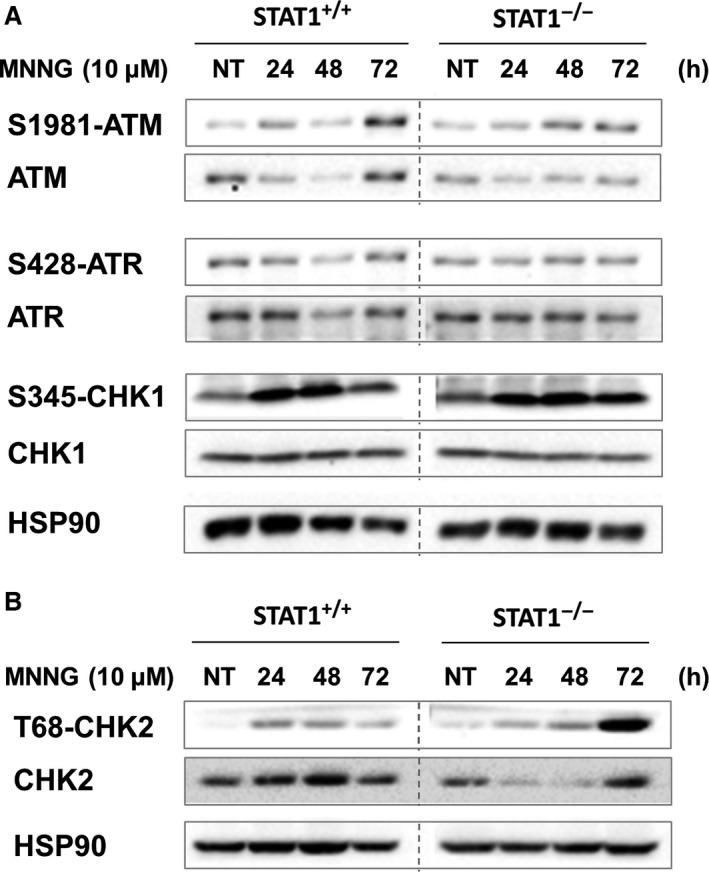
Expression and activation status of early DNA damage effectors according to STAT1. Levels of expression and phosphorylation of ATM, ATR, and CHK1 (**A**) or CHK2 (**B**) after 1 hr treatment or not (NT) with 10 μM MNNG were analysed by western blotting at the indicated times. The membranes were subsequently probed with anti‐phosphorylated forms and anti‐total forms antibodies. Typical blots out of three experiments are shown. STAT1^+/+^ and STAT1^−/−^ panels are cut from the same western blot.

Since the MMR system is necessary for the repair of MNNG‐induced DNA damage, we thus studied whether STAT1 deficiency would impair expression of key components of this system. Neither STAT1 deficiency nor MNNG treatment altered the expression of MLH1 and MSH2 (Fig. S3). We then analysed whether MNNG treatment might allow the activation of early DNA damage effectors such as ATM/CHK2 or ATR/CHK1 in our model. Western blot analysis of ATM, ATR, CHK1 showed only mild differences in the level of expression or in the activation of these proteins between STAT1^+/+^ and STAT1^−/−^ cells following MNNG exposure (Fig. 4A). In marked contrast, we observed a persistent increase in activation of CHK2 through a 72 hrs period in STAT1^−/−^ cells compared to STAT1^+/+^ cells (Fig. 4B).

### STAT1 is necessary to the recruitment of c‐Abl to p53/DNA complex following MNNG treatment

**Figure 5:**
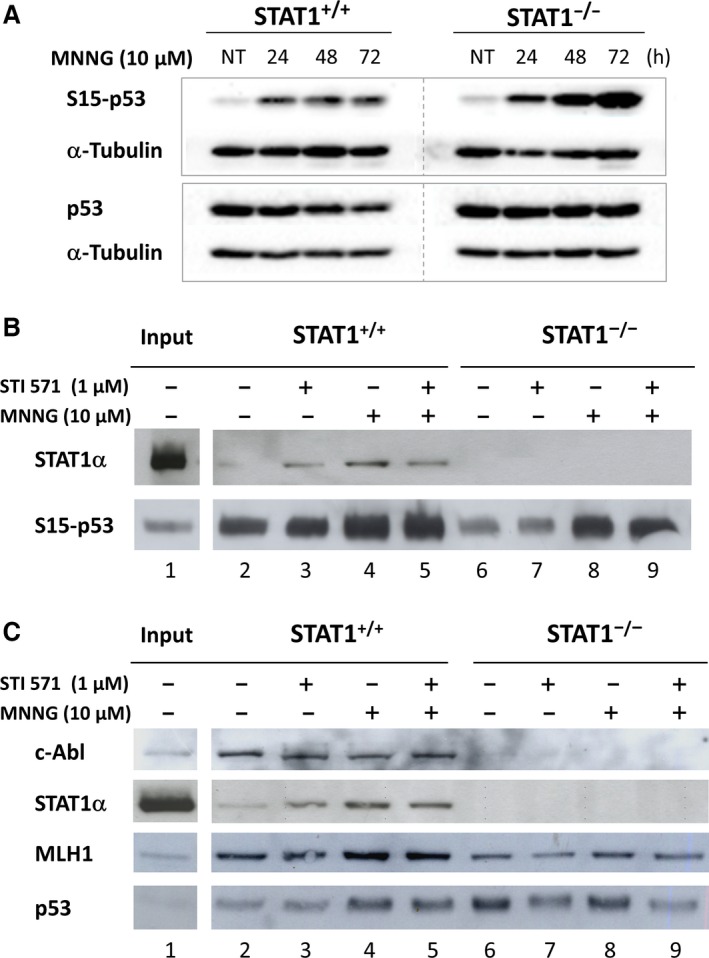
Recruitment in p53‐activated/DNA complex following MNNG treatment depends on STAT1. (**A**) Western blot analysis for the indicated proteins at various times after treatment of the cells or not (NT) with MNNG (1 hr exposure; 10 μM). S15‐p53 and p53 were analysed in two individual western blots. This experiment was repeated four times. STAT1^+/+^ and STAT1^−/−^ panels are cut from the same western blot. (**B** and **C**) STAT1^+/+^ and STAT1^−/−^ cells were treated (+) or not (−) with 10 μM MNNG for 1 hr, in the presence (+) or not (−) of STI571 inhibitor (1 μM). 72 hrs following treatment, nuclear extracts were incubated with a biotinylated oligonucleotide containing the target canonical consensus sequence for activated p53. Complexes bound to activated‐p53 (S15) were analysed by western blotting using antibodies recognizing the indicated proteins. A total of 10 μg of the nuclear extracts were also loaded as a control of expression of the various proteins in STAT1^+/+^ cell extracts (input). A typical experiment out of two is shown.

Since p53 is a substrate of the CHK2 kinase 31, we analysed the kinetics of p53 activation using western blotting in our model. We observed a stronger activation of p53 in STAT1^−/−^ cells compared to STAT1^+/+^ cells following exposure to MNNG (Fig. 5A). p53 has already been described to interact physically either with MMR complexes or STAT1 in different contexts. Therefore, we investigated p53 partners in our model using a p53 ODN pull down assay. We observed the recruitment of STAT1 to serine‐15‐phosphorylated p53 complex in STAT1^+/+^ cells whether MNNG‐treated or not (Fig. 5B and C). Furthermore, studying other known partners, we also observed in the complex the constitutive presence of c‐Abl and MLH1 in STAT1^+/+^ cells (Fig. 5C). These recruitments were not modified by MNNG treatment of STAT1^+/+^ cells (Fig. 5C, lanes 2 *versus* 4). Using STI571, a specific inhibitor of c‐Abl, we ruled out the implication of c‐Abl kinase activity on the recruitment of MLH1, STAT1 and c‐Abl itself to the p53‐DNA complex (see Fig. 5B and C, lanes 3 *versus* 5). Strikingly, the complex formed with p53 in STAT1^−/−^ cells contained MLH1 but not c‐Abl (Fig. 5C, lanes 6 to 9) demonstrating that STAT1 was prevalent to the recruitment of c‐Abl in the complex.

### STI571 treatment protects STAT1‐expressing cells from MNNG‐induced cytotoxicity

**Figure 6:**
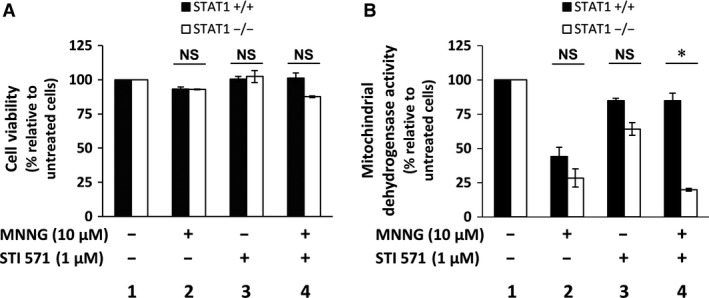
STI571 impacts on MNNG‐induced cytotoxicity according to STAT1 expression. STAT1^+/+^ and STAT1^−/−^ cells were exposed (+) or not (−) to 10 μM MNNG for 1 hr in the presence (+) or not (−) of 1 μM STI571 inhibitor. Viability was studied after 72 hrs by Trypan blue vital coloration (**A**) and cell dehydrogenase activity by MTS assay (**B**). Results are plotted as the percentage relative to untreated cells. Data points are the mean of triplicates. Error bars represent the standard deviation. These experiments were repeated twice and statistical significance was determined using the Wilcoxon signed‐rank test (NS, non‐significant; *P < 0.05).

We then investigated a possible role for c‐Abl tyrosine kinase activity in the decrease in cell viability and metabolic activity and cell induced by MNNG using the inhibitor STI571. STI571 alone had limited effects on viability and metabolic activity (Fig. 6A and B, lane 3). *N*‐methyl‐*N*′‐nitro‐*N*‐nitrosoguanidine exposure decreased the metabolic activity independently of STAT1 expression (Fig. 6B, lane 2 and Fig. 1C right). Combined exposure to MNNG and STI571 restored the metabolic activity in STAT1^+/+^ cells only (Fig. 6B, lane 4). Taken together, these results suggest that MNNG‐induced cytotoxicity involves c‐Abl kinase activity depending on the presence of STAT1.

### STAT1 protects cells from long‐term cytotoxicity of MNNG

**Figure 7:**
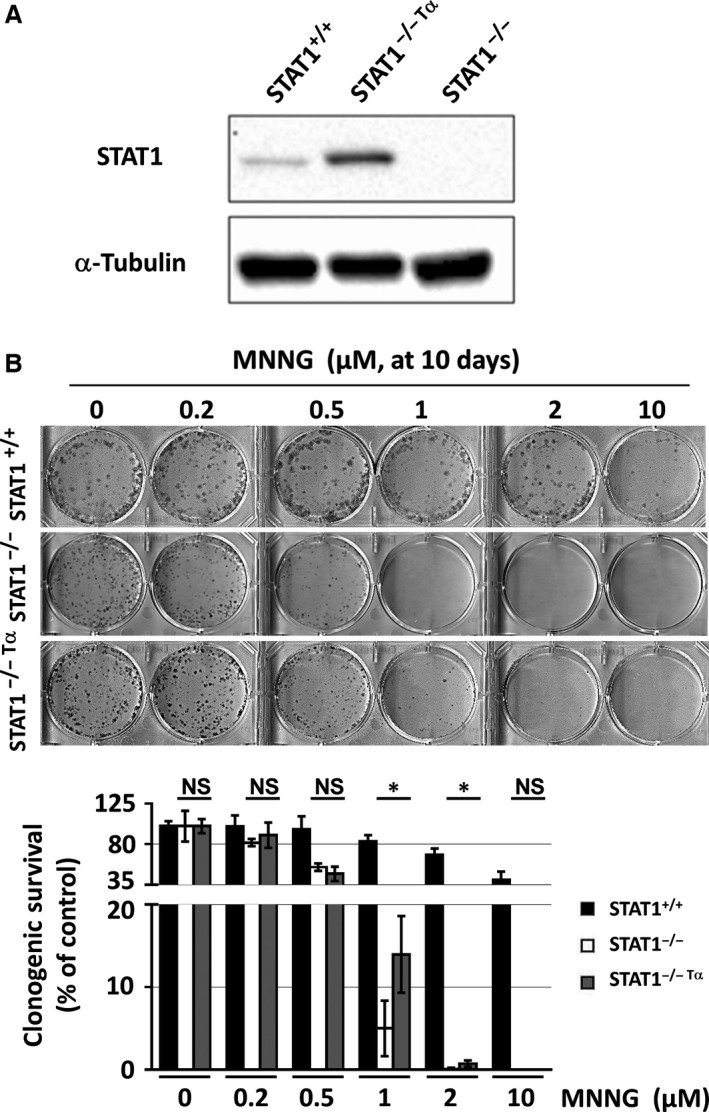
Effect of *de novo* expression of STAT1 alpha isoform on long‐term survival following MNNG exposure. (**A**) Total extracts from STAT1^+/+^, STAT1^−/−^ and STAT1^−/−Tα^ cell lines were analysed by western blotting using antibodies recognizing the indicated proteins. A typical blot out of five is shown. (**B**) Clonogenic survival was analysed in the indicated cell lines at 10 days following a 1 hr exposure to the indicated doses of MNNG. A typical clonogenic assay representative of five independent experiments is shown. Percentage of colonies containing 50 cells or more was evaluated relative to untreated control. Statistical significance was determined by paired *t*‐test (*P < 0.05).

We confirmed the effect of STAT1 upon MNNG treatment using the clonogenic survival assay in STAT1^−/−^ cells transfected with the alpha isoform of STAT1 (STAT1^−/−Tα^). STAT1^−/−^ transfected cells were able to form colonies up to 2 μM MNNG treatment. Therefore, re‐expression of STAT1 restored an intermediate sensitivity to MNNG as compared to STAT1^+/+^ and STAT1^−/−^ (Fig. 7A and B). These results confirm that, in our model, STAT1 protects cells from long‐term cytotoxicity of MNNG treatment.

## Discussion

We report that the transcription factor STAT1 is a key modulator of cellular response to DNA alkylation caused by the S_N_1 methylator MNNG. We provide evidence that the presence of STAT1 is critical for DNA repair and survival following MNNG exposure, attested by: (i) limited activation of histone H2AX, (ii) subsequent cell cycle progression and (iii) long‐term clonogenic survival. Our results also suggest that STAT1 modulates MNNG‐induced cytotoxicity through the assembly of STAT1, c‐Abl and MLH1 to p53‐DNA complex, and the involvement of the tyrosine kinase activity of c‐Abl.

In our model, the physiological repair of base‐pair mismatches seems independent of STAT1 as attested by the absence of microsatellite instability in STAT1^−/−^ cells. Moreover, the level of expression of MLH1 and MSH2 was independent on the presence of STAT1. In a genotoxic context, Stojic *et al*. have shown in another fibroblastic model that following a 10 μM MNNG exposure, the cellular response remains dependent on the MMR system 32. In accordance with delayed activation of the MMR system by S_N_1 methylators 33, we observed a delayed decrease in metabolic activity after exposure to MNNG with similar kinetics in STAT1^+/+^ and STAT1^−/−^ cells, whereas it appeared earlier following cisplatin treatment. We also observed a G_2_/M accumulation in both STAT1^+/+^ and STAT1^−/−^ cells following treatment with MNNG. Thus, since STAT1 has been shown to regulate G1/S cell cycle arrest by inverse modulation of cyclin D1, and p21^Cip1^ or p27^Kip1^ 34, our results favour a STAT1‐independent G_2_/M arrest.

However, we observed that STAT1 expression is associated with increased resistance to MNNG. Extensive MNNG‐induced O^6^MeG:T mispairing generates double strand breaks leading to histone H2AX phosphorylation 6. In STAT1^−/−^ cells, MNNG induced more pronounced histone H2AX activation and subsequent sustained CHK2 and p53 phosphorylation, a persistent G_2_/M accumulation and an absence of long‐term survival, suggesting impaired DNA repair leading to cell death. In marked contrast, in STAT1^+/+^ cells, MNNG induced limited H2AX, CHK2 and p53 phosphorylation, and a transient G_2_/M accumulation, in line with an efficient DNA repair allowing clonogenic survival. Interestingly, *de novo* overexpression of the alpha isoform of STAT1 in STAT1^−/−^ (STAT1^−/− Tα^) cells led to an intermediate sensitivity to MNNG (Fig. 7). This difference in sensitivity might be due to the absence of the beta isoform of STAT1 in STAT1^−/− Tα^ cells. Indeed, previous work in our laboratory demonstrated differential mechanisms between the alpha and beta isoforms of STAT1 leading to apoptosis in response to genotoxic stress 35.

Altogether, our results showed that STAT1 is required for repair of DNA double strand breaks, subsequent cell cycle progression and survival following genotoxic stress induced by MNNG. This is in keeping with previous studies that associated STAT1 with resistance to different types of DNA damage such as irradiation 36, 37 or exposure to doxorubicin 38.

To further investigate the potential role of STAT1 in MMR signalling, we analysed its potential partners, p53 and c‐Abl, two pivotal signalling factors involved in cell fate orientation following DNA damage 39, 40. Youlyouz‐Marfak *et al*. have observed interactions between STAT1/c‐Abl and p53/STAT1 in a lymphoblastoid cell line treated by doxorubicin 25. Both STAT1 and c‐Abl have also been described as stabilizing p53 and potentiating its transcriptional and biological activities following DNA damage 23, 41. Thus, we analysed whether the protective effect of STAT1 following MNNG exposure could depend on interactions between STAT1 and putative partners altogether with the transcriptionally active fraction of p53 using a pull down assay. Importantly, we observed a novel interaction between MLH1 and p53 that was independent on the presence of STAT1. This result can be related to the previously described interactions between p53 and MSH2 42 and between MLH1 and c‐Abl 10, and we provide further evidence for the participation of p53 to a multiproteic MMR complex. Besides the implication of p53 in the transcriptional control of *MLH1*,* PMS2* and *MSH2* genes 43, this p53/MLH1 complex formation could additionally contribute to the modulation of MLH1‐induced MMR signalling by p53.

Our oligonucleotide pull down experiments indicated the presence of STAT1, c‐Abl, MLH1 and p53 at the p53 response element. Importantly, STAT1 modulated the protein composition of this complex since c‐Abl was incorporated into the complex in the presence of STAT1 only. The absence STAT1 and therefore of c‐Abl at the p53‐response element, was associated with an accumulation of double strands breaks, sustained G_2_/M arrest and poor clonogenic survival. Since both STAT1 and c‐Abl are known regulators of p53, we speculate that the composition of this complex may affect the panel of p53 transcriptional targets following DNA damage. These results suggest a new STAT1/c‐Abl regulation of p53 activity. The regulation by STAT1 and/or c‐Abl of p53 transcriptional activity on its target genes including regulators of G_2_/M arrest such as GADD45α 44 or 14‐3‐3σ 45 will require further investigation.

To delineate the role of c‐Abl in the cellular response to MNNG in our model, we inhibited its tyrosine kinase activity with the inhibitor STI571. Our oligonucleotide pull down results indicated that the interaction between c‐Abl and p53 does not require c‐Abl kinase activity, confirming other data 46. Importantly, we demonstrated that the mechanism of toxicity induced by MNNG treatment varies according to the presence of STAT1. The toxicity of MNNG depended on c‐Abl‐tyrosine kinase activity in the presence of STAT1 only. Our results are in accordance with the previously described effect of STI571 that has been shown to abolish G_2_/M arrest and to favour cell survival 16, 17. They are also in agreement with recent data by Udden *et al*. demonstrating that c‐Abl kinase activity modulates p53‐despendent cell fate decision in response to DNA damage 47.

A possible model of cellular response to MNNG, based on data from both our group and other reports, relies on an ATM/CHK2/p53 signalling pathway driven by STAT1 and controlling cell cycle reentry. One of the targets would be the activation of p53 through the involvement of a complex comprising STAT1/c‐Abl/MLH1/p53 allowing DNA repair, subsequent cell cycle progression and cell survival. We speculate that besides c‐Abl, MLH1 and STAT1, other partners of p53 might constitute a larger multiproteic complex following DNA damage. Considering the multilevel interplay between its components, such a complex may function as a platform integrating several signals, eventually orienting cell fate following genotoxic stress. Putative influence of STAT1 deficiency on additional DNA methylation repair systems such as MGMT would deserve further investigation.

Our data confirm previous reports stating that inhibitors of c‐Abl such as STI571 should not be combined with alkylating agents, which depend on MMR functional response for efficacy 16, 17. Finally, our results indicate that STAT1 is a modulator of the signalling pathway activated by the MMR system and is essential for DNA repair and cell survival following genotoxic stress induced by alkylating agents. The ability of STAT1 to favour resistance to MNNG suggests that targeting the STAT1 pathway may be a therapeutic option for enhancing the efficacy of S_N_1 alkylating agent‐based chemotherapy.

## Conflict of interest

The authors confirm that there are no conflicts of interest.

## Supporting information


**Figure S1** Western blot analysis of STAT1 and Y701‐STA1 expression levels in STAT1^+/+^ and STAT1^−/−^ cells following treatment with MNNG.

**Figure S2** Short‐term mitochondrial dehydrogenase activity following cisplatin exposure is dependent on STAT1.

**Figure S3** Western blot analysis of MLH1 and MSH2 expression levels in STAT1^−/−^ and STAT1^+/+^ cells.

**Table S1** Size of microsatellite markers in STAT1^+/+^ and STAT1^−/−^ cell lines.
