# Supplementary figures and images for: Cellular response to alkylating agent MNNG is impaired in STAT1‐deficients cells
Source: J Cell Mol Med. 2016 Jul 27;20(10):1956–65. doi: 10.1111/jcmm.12887 (PMC5020624; doi:10.1111/jcmm.12887)

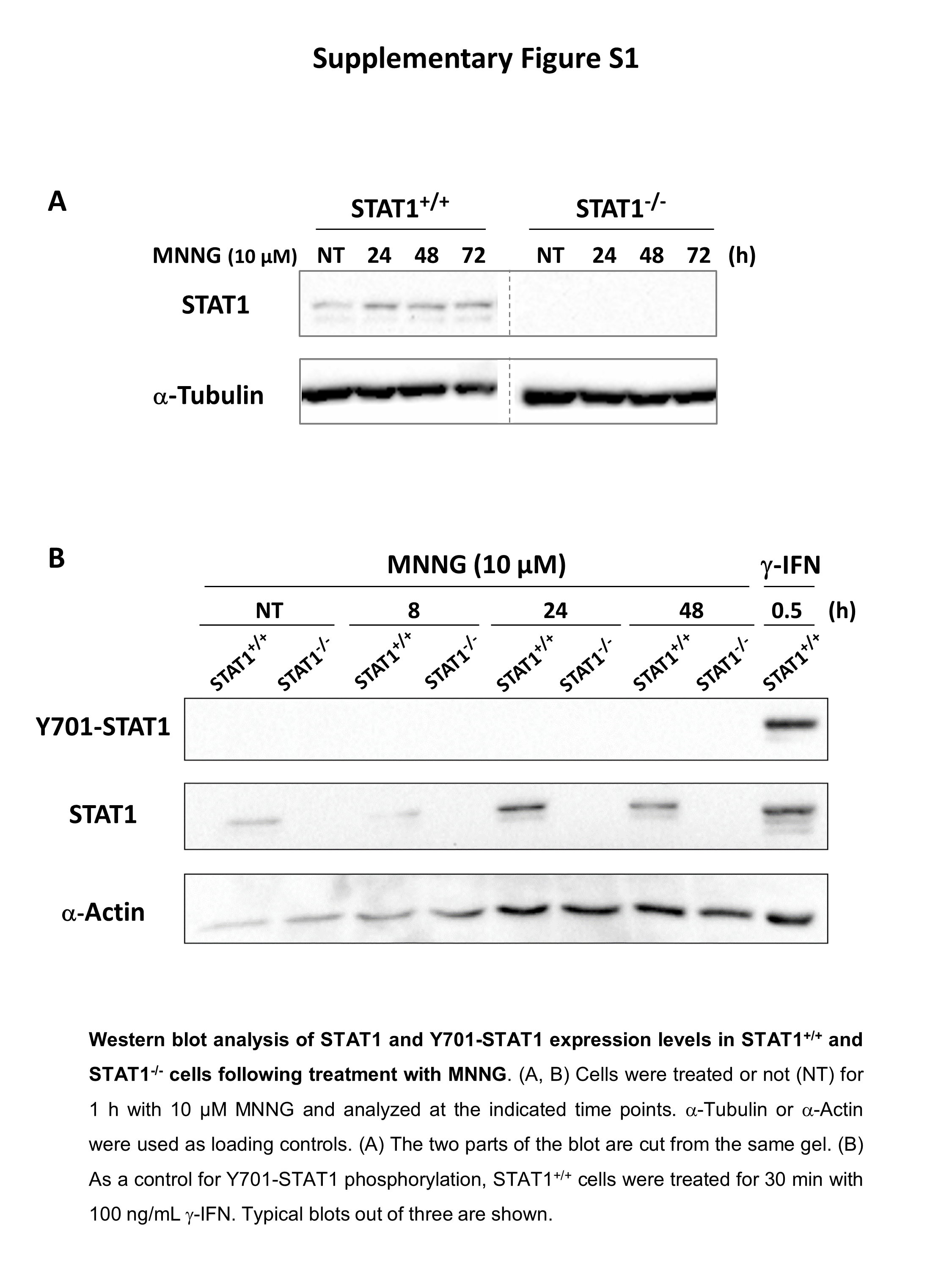

Supplement: Supplementary file 1 — Figure S1 Western blot analysis of STAT1 and Y701‐STA1 expression levels in STAT1+/+ and STAT1−/− cells following treatment with MNNG. [file JCMM-20-1956-s001.tif]

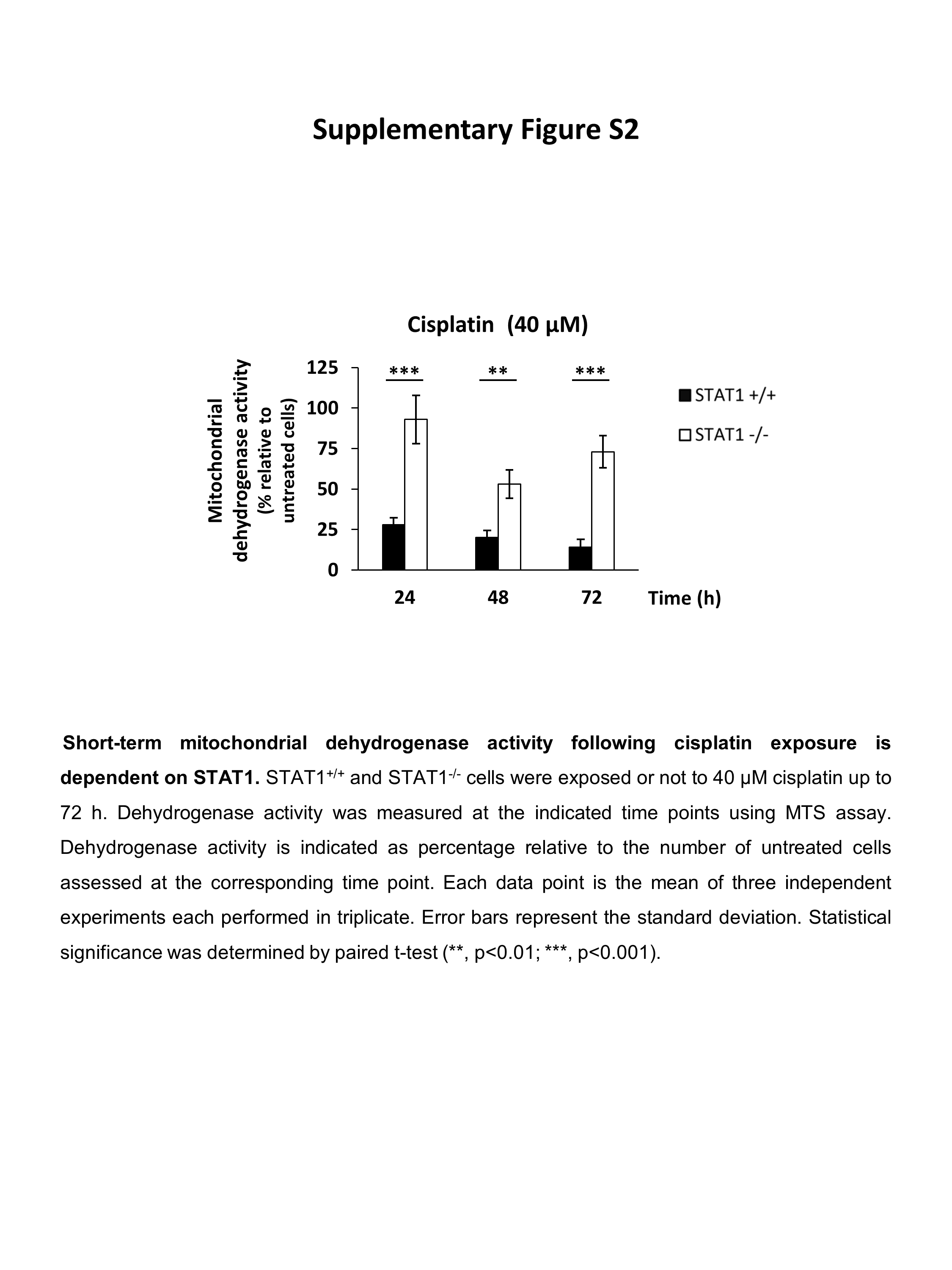

Supplement: Supplementary file 2 — Figure S2 Short‐term mitochondrial dehydrogenase activity following cisplatin exposure is dependent on STAT1. [file JCMM-20-1956-s002.tif]

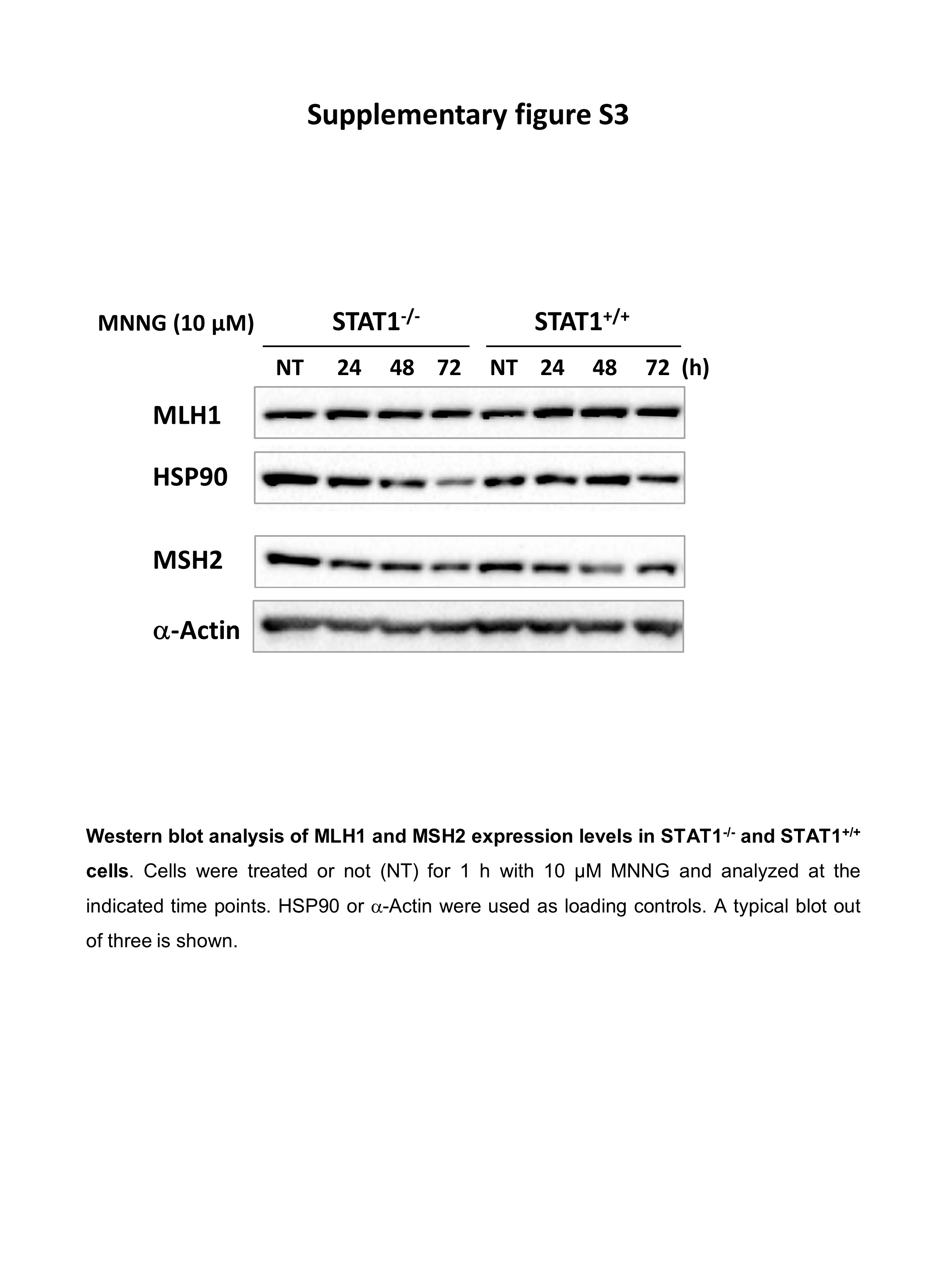

Supplement: Supplementary file 3 — Figure S3 Western blot analysis of MLH1 and MSH2 expression levels in STAT1−/− and STAT1+/+ cells. [file JCMM-20-1956-s003.tif]

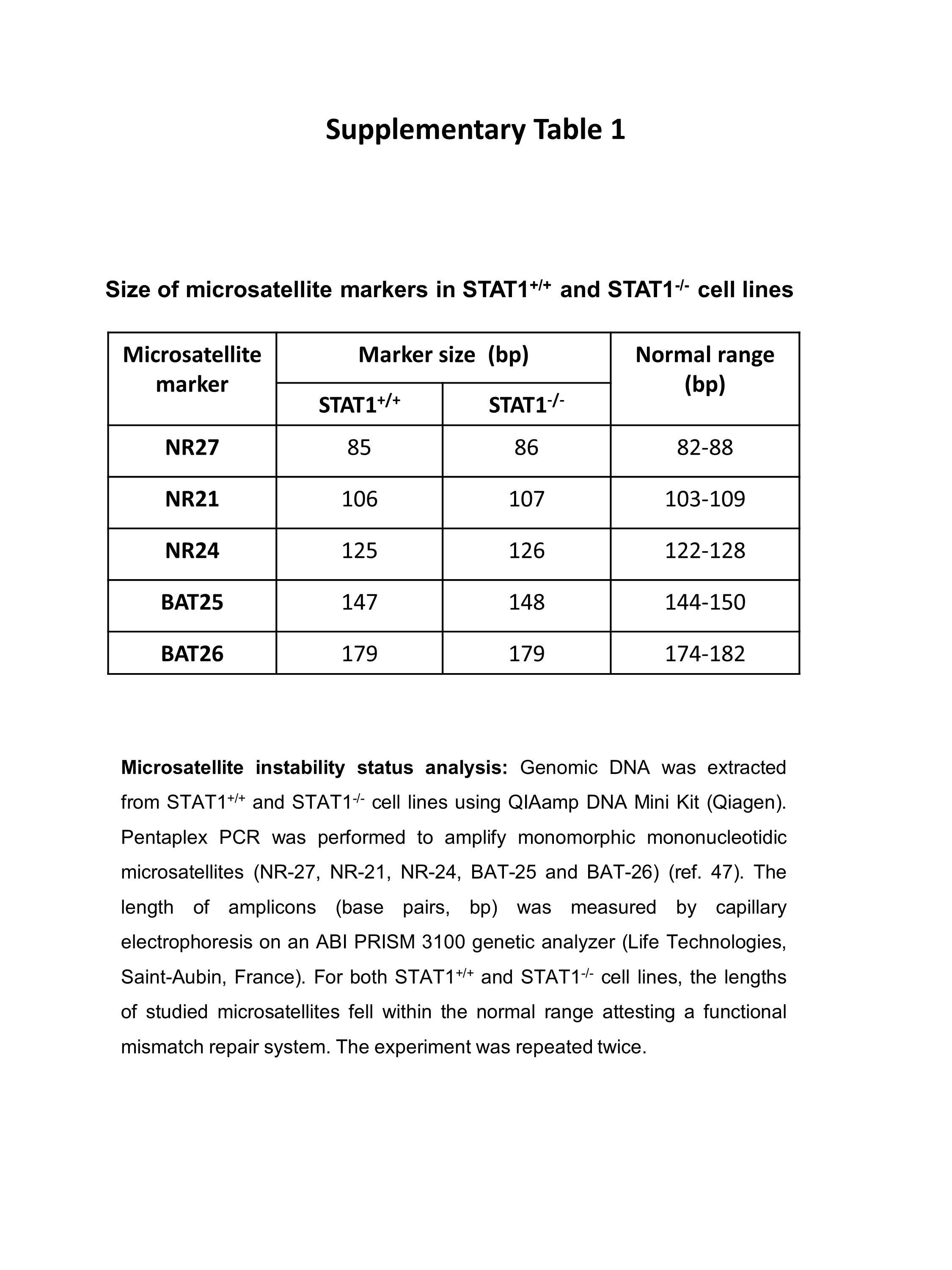

Supplement: Supplementary file 4 — Table S1 Size of microsatellite markers in STAT1+/+ and STAT1−/− cell lines. [file JCMM-20-1956-s004.tif]
